# Supplementary material for: The Influence of Recommendation of Medical and Non-Medical Authorities on the Decision to Vaccinate against Influenza from a Social Vaccinology Perspective: Cross-Sectional, Representative Study of Polish Society
Source: Vaccines (Basel). 2023 May 17;11(5):994. doi: 10.3390/vaccines11050994 (PMC10221997; doi:10.3390/vaccines11050994)
Supplement: Supplementary file 1 [file vaccines-11-00994-s001.zip › vaccines-2382870-supplementary.pdf]

Supplementary Materials

The Questionnaire (translated form polish to English)

TRUST IN MEDICAL AND NON-MEDICAL AUTHORITIES ON THE ISSUE OF INFLUENZA VACCINATION

Dear Sirs,

The following questionnaire is the basic stage of a research project aimed at finding out the attitudes of the Polish public toward influenza vaccination.

Participation in the study is voluntary.

The survey is confidential, and the data obtained from the survey will be analyzed collectively. Any identification of responses with a specific person completing the survey questionnaire will be impossible.

If you choose to participate in the survey, please proceed to the next section, if not, please complete the survey.

1. **If your doctor recommended that you be vaccinated against a disease, then:**
    a. I would vaccinate/vaccinate without hesitation
    b. I would/should vaccinate, but I would/should consult another professional beforehand, e.g., another doctor or nurse.
    c. I would/should vaccinate myself, but before that I would have to/should read about the vaccination on the Internet
    d. A doctor's recommendation would not have affected my decision to vaccinate

2. **To what extent would the opinion/opinion of the following people matter if you wanted to get vaccinated against influenza?** *Scale from 1 to 5, where 1 means "very low influence" and 5 means "very high influence".*
    a. Nurse
    b. Doctor
    c. Pharmacist in a pharmacy
    d. Someone from the family/friends

3. **Have you been vaccinated against COVID-19?**
    a. Yes, I have, the first doses during the pandemic and the booster dose(s)
    b. Yes, I have, but only with the first doses during the pandemic, no booster doses
    c. I have not

4. **Is the COVID-19 pandemic and the related discussion on vaccination:**
    a. Strengthened your confidence in vaccination
    b. You have weakened your confidence in vaccination
    c. You have not changed your approach to vaccination

5. **What is your attitude toward vaccinations in general - regardless of what diseases they are given against and to whom they are given (children, adults)?**
   a. Strong vaccine supporter
   b. Moderate vaccine supporter
   c. Moderate vaccine opponent
   d. Strong vaccine opponent
